# Fermented *Protaetia brevitarsis* Larvae Alleviates High-Fat Diet-Induced Non-Alcoholic Fatty Liver Disease in C57BL/6 Mice via Regulation of Lipid Accumulation and Inflammation

**DOI:** 10.4014/jmb.2409.09025

**Published:** 2025-02-10

**Authors:** Hyo Lim Lee, Jong Min Kim, Min Ji Go, Han Su Lee, Ju Hui Kim, In Young Kim, Geum-Su Seong, Ho Jin Heo

**Affiliations:** 1 Division of Applied Life Science (BK21), Institute of Agriculture and Life Science, Gyeongsang National University, Jinju 52828, Republic of Korea; 2 Korea Food Research Institute (KFRI), Wanju Zipcode, Republic of Korea

**Keywords:** *Protaetia brevitarsis* larvae, type 2 diabetes mellitus, hepatoxicity, oxidative stress, mitochondrial function, AMPK

## Abstract

Non-alcoholic fatty liver disease (NAFLD), characterized by hepatic steatosis and hepatitis, is the most frequently encountered complication of type 2 diabetes mellitus (T2DM). Due to its hepatoprotective, anti-obesity, antioxidant, and anti-inflammatory effects, *Protaetia brevitarsis* (*P. brevitarsis*) larvae have been used as traditional medicine to treat liver diseases since ancient times. Therefore, this study was conducted to confirm the positive effect of fermented *P. brevitarsis* larvae (FPB) on NAFLD. The results showed that high-fat diet (HFD)-induced dysglycemia was improved by treatment with FPB as determined by testing for fasting blood glucose and oral glucose tolerance. The weight of liver and white adipose tissue and the levels of serum lipid, hepatotoxicity, and nephrotoxicity indicators were reduced by FPB. In addition, oxidative stress and mitochondrial dysfunction caused by HFD were improved by FPB. In a similar manner, HFD-induced hepatic steatosis was prevented by FPB through regulation of the AMP-activated protein kinase pathway and serum lipid profile. HFD-induced hepatitis and apoptosis were ameliorated by FPB via the nuclear factor-kappa B pathway and the B-cell lymphoma 2 protein family. In conclusion, this study suggests the potential for application of FPB as a prophylactic agent for treatment of NAFLD through suppression of lipid accumulation and inflammation in the liver.

## Introduction

Nonalcoholic fatty liver disease (NAFLD) encompasses a broad spectrum of conditions, including hepatic steatosis, nonalcoholic steatohepatitis (NASH), and cirrhosis, making it the most common liver disease, with a global prevalence of 30% [1]. According to a recent study, the cause of NAFLD is a combination of fat accumulation, lipotoxicity, oxidative stress, inflammation, insulin resistance (IR), and mitochondrial dysfunction [2]. In particular, the prevalence of obesity and type 2 diabetes mellitus (T2DM) contributes to NAFLD by causing lipid metabolism disorders [2]. It is considered the strongest risk factor for faster progression to NASH, advanced fibrosis, and cirrhosis [3]. Elevated blood sugar levels due to consumption of a high-fat diet (HFD) cause abnormalities of glucose and lipid metabolism in hepatocytes and eventually lead to hepatic steatosis [4]. Therefore, the prevention and improvement of NAFLD can be promoted by regulating glucose and lipid metabolism in the liver. AMP-activated protein kinase (AMPK) plays an important role in bioenergy metabolism [5]. When AMPK is activated in the liver, it inhibits the synthesis of fatty acids and cholesterol and boosts fatty acid oxidation [5]. Activated AMPK promotes ATP production and inhibits cholesterol biosynthesis process by phosphorylating enzymes related to energy metabolism, such as HMG-CoA reductase (HMGCR) [6]. Therefore, the exploration of bioactive compounds targeting AMPK is considered an effective strategy to improve hepatic steatosis [6].

Excessive accumulation of lipids in liver cells acts as a cytotoxic agent, causing dysfunction and apoptosis of cells. This is called lipotoxicity, and the resulting oxidative stress and inflammatory response is recognized as a significant risk factor for NAFLD [7]. Hyperglycemia caused by high fat intake promotes the production of reactive oxygen species (ROS) and depletes intracellular antioxidant substances such as superoxide dismutase (SOD) and glutathione (GSH), increasing oxidative stress [8]. As a result, excessive oxidative stress causes mitochondrial dysfunction through changes in the mitochondrial membrane potential (MMP) and activates apoptosis signals [8]. Meanwhile, HFD-induced hyperglycemia acts as a signal to activate innate immunity through Toll-like receptor (TLR) by activating the protein kinase C (PKC) pathway [9]. Afterward, it mediates the inflammatory response by activating nuclear factor-κB (NF-κB) through an intracellular signaling system and increasing pro-inflammatory cytokines such as interleukin (IL)-1β and tumor necrosis factor (TNF)-α and inducible enzymes such as cyclooxygenase (COX)-2 and nitric oxide synthase 2 [10]. Therefore, regulating antioxidant systems and inflammation through the TLR-4/NF-κB pathway may be an essential strategy to prevent the development of NASH caused by hepatic steatosis.

Edible insects have been utilized in traditional medicine for centuries, and due to their high protein content, they are considered nutritionally superior [11]. In particular, *Protaetia brevitarsis* (*P. brevitarsis*) larvae, a coleopteran insect, is reported to have various bioactivities such as anti-obesity, antioxidant, and anti-inflammatory effects as a functional material [12-14]. Moreover, *P. brevitarsis* larvae have been used as traditional medicine for hepatic diseases since ancient times [15]. Research on the various physiological effects of *P. brevitarsis* larvae has been reported, but development is limited as food due to negative perceptions of insects [16]. On the other hand, fermentation as a processing technology can not only change the perception of edible insects but also enhance bioactive compounds such as polysaccharides, peptides, fatty acids, polyphenols, alkaloids, and carotenoids connected in edible insects [16-18]. Indeed, in our prior study, we analyzed the amino acid contents in fermented *P. brevitarsis* larvae (FPB) and confirmed that they were higher than those in the aqueous, ethanol, and methanol extracts of *P. brevitarsis* larvae [19]. FPB is rich in essential amino acids such as lysine, methionine, and threonine, which can help restore liver function by contributing to the regeneration of liver cells and protein synthesis. Despite this, research on the liver protective effect of *P. brevitarsis* larvae fermented with beneficial bacteria such as *Bacillus subtilis* (*B. subtilis*) is lacking. Moreover, although great progress has been made in understanding the pathogenesis of NAFLD, there are still no internationally approved pharmacological treatments that can reliably reverse NAFLD [3]. Thus, the current study aimed to prove the improvement effect of FPB in HFD-induced NAFLD mice.

## Materials and Methods

### Sample Preparation

The FPB powder product used in the experiment was supplied by HMO Health Dream (Taean, Republic of Korea). FPB was prepared in the same method as in our previous study [19]. Briefly, *P. brevitarsis* larvae were hydrolyzed with protease along with papain and bromelain and then fermented using *B. subtilis* KCTC 1428BP (1×10^5^ CFU/ml) at 30°C for 48 h. After, FPB were evaporated under reduced pressure and lyophilized. The samples were stored at 20°C until further use.

### Cell Viability

To determine the cell viability, it was assessed using the 3-(4,5-dimethylthiazol-2-yl)-2,5-diphenyltetrazolium bromide (MTT) method [20]. HepG2 cells were seeded on 96-well plate at a density of 1 × 10^4^ cells/well for 24 h. Cells were treated to different concentrations of FPB for 30 min, and then 50 mM glucose was added. After 24 h incubation, the MTT stock solution (5 mg/ml) was reacted for 3 h and, the media were removed. The produced MTT formazan crystals were dissolved in dimethyl sulfoxide, and measured at wavelengths of 570 and 620 nm using a microplate reader (Epoch 2, BioTek Instruments, Inc., USA).

### Intracellular Oxidative Stress

To detect the intracellular oxidative stress level, it was assessed using the 2,7-dichlorodihydrofluorescein diacetate (DCF-DA) method [21]. HepG2 cells were seeded on 96-well black plate as 1 × 10^4^ cells/well for 24 h. Cells were exposed with different concentrations of FPB for 30 min, and then 50 mM glucose was added. After 24 h incubation, the DCF-DA stock solution (10 μM) was reacted for 50 min. The level of ROS was measured using a fluorescence microplate reader (Infinite 200, Tecan Co., Switzerland) at 485 nm excitation wavelength and 530 nm emission wavelength.

### Animal Experiment Design

**Fig. 1:**
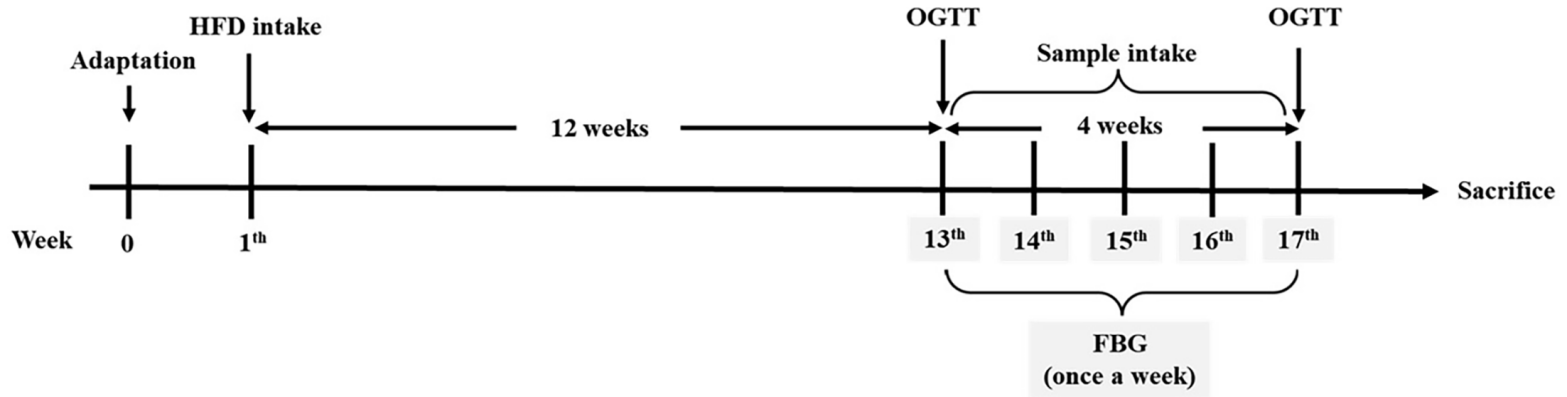
Scheme of animal experiment design.

A total of 60 C57BL/6 male mice (4 weeks old, *n* = 15 per group: mitochondrial test, n =5; *in vivo* test, n =10 (serum biomarkers and antioxidant parameter tests, *n* = 7; western blotting, *n* = 3)) were purchased from Samtako (Republic of Korea). The mice were housed at a constant temperature (23 ± 1°C) and humidity (55%) with a 12 h light-dark cycle and had free access to food and water. Mice were randomly assigned to four groups: control group (fed a normal diet), HFD group (fed an HFD), FPB 50 group (fed an HFD + FPB 50 mg/kg of body weight), and FPB 100 group (fed an HFD + 100 mg/kg of body weight). Body weight and food consumption were measured every week during conduct of the experiment. HFD was provided for 16 weeks. Mice were fed an HFD for 12 weeks to induce diabetes. Fasting blood glucose (FBG) and oral glucose tolerance test (OGTT) were performed for selection of mice with hyperglycemia of FBG level of 140 mg/ml or higher in the 13^th^ week (Fig. S1). FPB dissolved in drinking water was then administered to selected diabetic mice by oral gavage for four weeks, and measurement of fasting blood sugar was performed every week during this period. The scheme of animal experimental designs is shown in Fig. 1. All animal study experiments were approved by the Institutional Animal Care and Use Committee (IACUC) of Gyeongsang National University guidelines (Certificate No. GNU-230214-M0027) on 14 February 2023.

### FBG and OGTT

Throughout the 4 weeks sample treatment period, FBG was measured every week on extracted blood samples from lateral tail vein. OGTT was performed at last week of sample treatment. After mice were fasted overnight, D-glucose dissolved in 0.85% NaCl was orally administered a dose of 2 g/kg of body weight. Blood glucose level was detected at 0, 15, 30, 60, 90 and 120 min using an Accu-Chek glucose meter (Roche Diagnostics, Switzerland). To determine the area under the curve (AUC) during OGTT results, it was calculated using trapezoidal rule.

### Biochemical Parameters in Serum

After OGTT, mice were fasted for 12 h and sacrificed using exposure to CO_2_. The blood sample was collected at the abdominal aorta to detected biochemical parameters. The collected blood samples were centrifuged at 10,000 ×*g* for 10 min at 4°C. The supernatant was immediately placed on ice and used for biochemicals.

The glutamic oxaloacetic transaminase (GOT), glutamine pyruvic transaminase (GPT), lactate dehydrogenase (LDH), blood urea nitrogen (BUN), creatine (CRE), total cholesterol (TCHO) and triglyceride (TG) were measured using clinical chemistry analyzer (Fuji dri-chem 4000i; Fuji film Co., Japan). Low-density lipoprotein cholesterol (LDLC) content and ratio of high-density lipoprotein cholesterol (HDLC) to TCHO (HTR) were calculated as follows [22]:

LDLC = TCHO -(HDLC + TG/5) and HTR (%) (HDLC/TCHO) ×100 (1)

### Tissue Preparation

After the mice were sacrificed, the liver, epididymal fat, perirenal fat, retroperitoneal fat, and mesenteric fat tissues were removed and rinsed with normal saline. The weight of fat tissues was immediately measured.

### Antioxidant Parameters

For antioxidant parameters analysis, the liver was minced with surgical scissors, divided into two tubes (one for reduced GSH analysis and the other for MDA and SOD analysis) and stored at -80°C.

**MDA content.** To assess lipid peroxidation in liver tissue, MDA content was measured. Liver tissues were homogenized with phosphate-buffered saline (PBS) and centrifuged at 2,500 ×*g* for 10 min. The supernatant was extracted and transferred to another e-tube. Then, it was mixed with 1% phosphoric acid and 0.67% thiobarbituric acid (TBA) and reacted at 95°C for 1 h. The absorbance of the reactant was measured at 532 nm. The MDA content in liver tissue were estimated according to a standard curve obtained 1,1,3,3-tetramethoxypropane.

**SOD activity.** To detect antioxidants in liver tissue, SOD activity was evaluated and detected using a commercial SOD kit (Dojindo Molecular Tech., USA) according to the manufacturer’s protocol. The SOD activity was measured by water-soluble tetrazolium (WST)-1 method [23].

**Reduced GSH level.** To confirm antioxidants in liver tissue, reduced GSH level was measured. Liver tissues were homogenized with 10 mM phosphate buffer with 1mM EDTA (pH 6.0) and centrifuged at 10,000 ×*g* for 15 min. The supernatant was obtained and mixed with 5% metaphosphoric acid and centrifuged at 2,000 ×*g* for 2 min. The supernatant was extracted and reacted with 0.26 M Tris-HCl (pH 7.6), 0.65 N NaOH, and o-phthaldialdehyde (OPT) for 15 min at room temperature by blocking the light. The fluorescence intensity of the reactant was measured at 320 nm (excitation wavelength) and 420 nm (emission wavelength).

### Mitochondrial Function

Isolation of mitochondria from liver tissues was performed as described previously [19]. Briefly, liver tissue was homogenized in isolation buffer (0.1% bovine serum albumin, 215 mM mannitol, 20 mM 2-(4-(2-hydroxyethyl)-1-piperazinyl)-ethane-sulfonic acid (HEPES) (Na^+^), and 75 mM sucrose, pH 7.2) containing 0.1% ethylene glycol tetra-acetic acid (EGTA) and centrifuged at 1,300 ×*g* for 5 min. The supernatant was transferred to a new tube and centrifuged at 13,000 ×*g* for 10 min. The obtained pellet was mixed with the isolation buffer containing 0.1%digitonin and centrifuged at 13,000 ×*g* for 15 min. The obtained pellets were resuspended in the isolation buffer and re-centrifuged at 10,000 ×*g* for 10 min. The final mitochondrial pellets were suspended in the isolation buffer and used for mitochondrial analysis.

**Mitochondrial ROS level.** To assess mitochondria function in liver tissue, mitochondrial ROS level was determined. The mitochondria extract from liver tissues was reacted with a KCl-based respiration buffer containing 125 mM KCl, 2 mM KH_2_PO_4_, 20 mM HEPES, 1 mM MgCl_2_, 500 μM EGTA, 2.5 mM malate, and 5 mM pyruvate. Then, the mixture was incubated with 25 μM DCF-DA for 20 min by blocking the light. After, the fluorescence intensity of the reactant was measured at 485 nm (excitation wavelength) and 530 nm (emission wavelength) for measurement of mitochondrial ROS level.

**MMP**. To estimate mitochondria function in liver tissue, MMP was detected. The mitochondria extract from liver tissues was incubated with 1 mM 1,1',3,3'-tetraethyl-5,5',6,6'-tetrachloroimidacarbocyanine iodide (JC-1) dye in mitochondrial isolation buffer containing 5 mM pyruvate and 5 mM malate. The mixture was reacted at room temperature for 20 min by blocking the light. Then, the fluorescence intensity was detected at 530 nm (excitation wavelength) and 590 nm (emission wavelengths).

**Mitochondrial ATP content.** To evaluate mitochondria function in liver tissue, ATP content was confirmed. ATP content was detected using ATP bioluminescence assay kit (Promega, USA) according to the manufacturer’s protocol. The ATP content was calculated using a standard curve.

### Western Blotting

To confirm the mechanism by which FPB improves HFD-induced liver function, western blot analysis was performed using only the control, HFD, and FPB100 groups. Liver tissues were homogenized in cell extraction buffer (Cell Signaling Tech., USA) containing 1% protease inhibitor cocktail (Quartett, Germany). The liver tissue lysates were centrifuged at 13,000 ×*g* for 10 min, and the protein concentration of supernatant was examined using a Brad-ford reagent (Bio-Rad, USA). The quantified samples were separated on sodium dodecyl sulfate-polyacrylamide gel and transferred onto a polyvinylidene fluoride membrane (Millipore, USA). The membranes were blocked with 5% skim milk solution for 1 h and washed three times with Tris-buffered saline containing 0.1%Tween 20 (TBST). The membranes were then incubated with respective primary antibodies (dilution of 1:1,000) overnight at 4°C, followed by washing three times with TBST and treated with the corresponding secondary antibodies (dilution of 1:3,000) at room temperature for 1 h. The intensity of protein bands was detected using an enhanced chemiluminescence detection reagent (Translab., Republic of Korea) and measurements were performed using an iBright CL1000 imager (Thermo Fisher Scientific, USA).

### Statistical Analysis

The results were analyzed using one-way analysis of variance followed by Tukey’s honest significant difference test by SAS program (Ver. 9.4 SAS Institute, USA). Data are showed as mean ± standard deviation. Statistical significance (*p* < 0.05) between the groups is indicated by lowercase letters in superscripts.

## Results

### Cytoprotective Effect of FPB on High Glucose-Induced HepG2 Cells

**Fig. 2:**
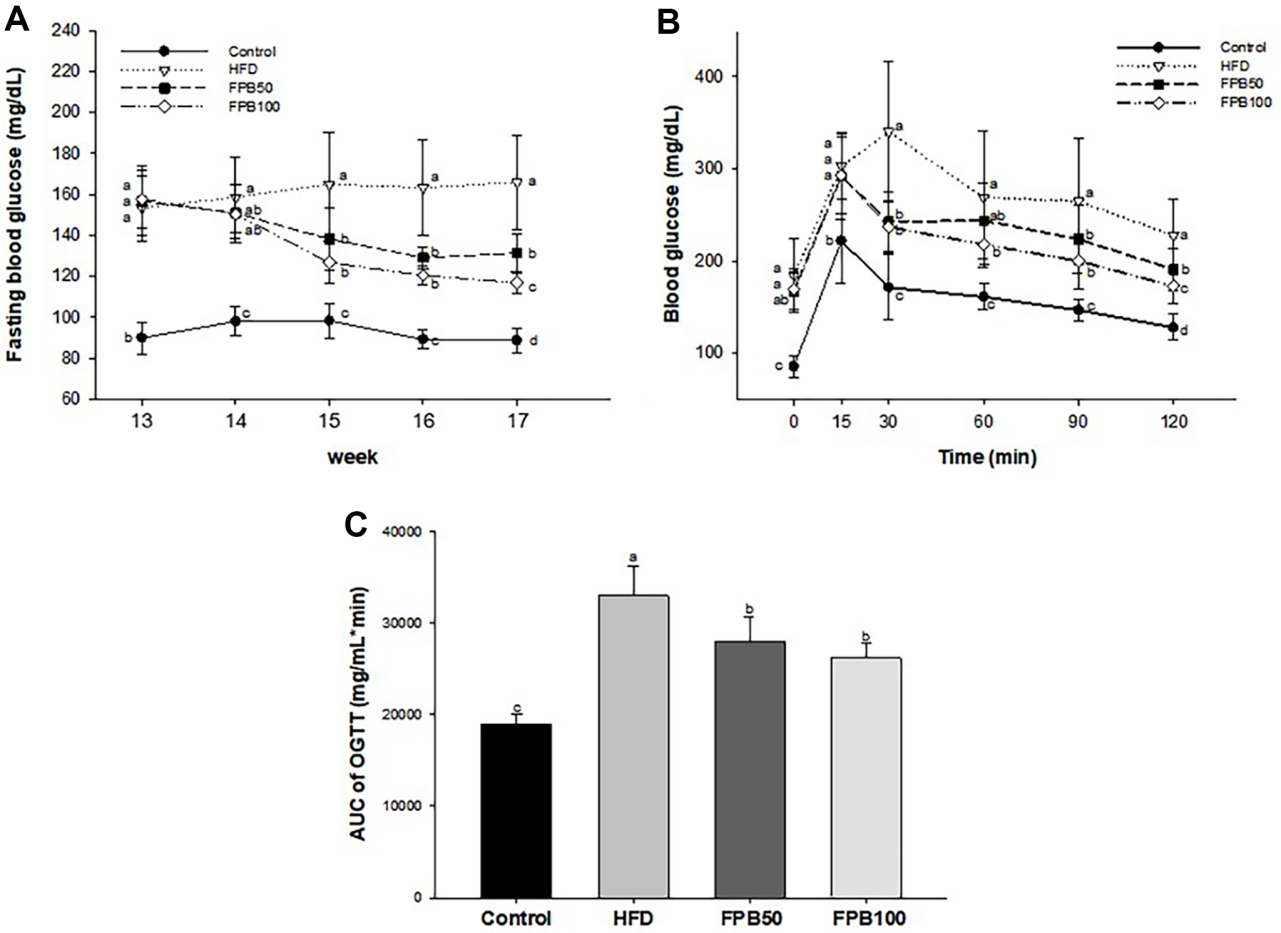
Protective effect of fermented *Protaetia brevitarsis* larvae (FPB) in high glucose-induced cytotoxicity in HepG2 cells. Cell viability (**A**) and oxidative stress (**B**). Results are expressed as mean ± SD (*n* = 5). Data were statistically considered at *p* < 0.05, and different lowercase letters represent statistical differences.

As shown in Fig. 2A, the cell viability of high glucose-treated group as negative group was decreased compared to control group. However, FPB treated groups were increased compared to negative control group at concentration of 50, 100, 200, and 500 μg/ml.

As shown in Fig. 2B, the intracellular oxidative stress of high glucose-treated group (136.66%) as negative group was increased compared to control group (100.00%). However, FPB treated groups were compared to negative control group at concentration of 50, 100, 200, and 500 μg/ml.

### Effect of FPB on FBG and OGTT

**Fig. 3:**
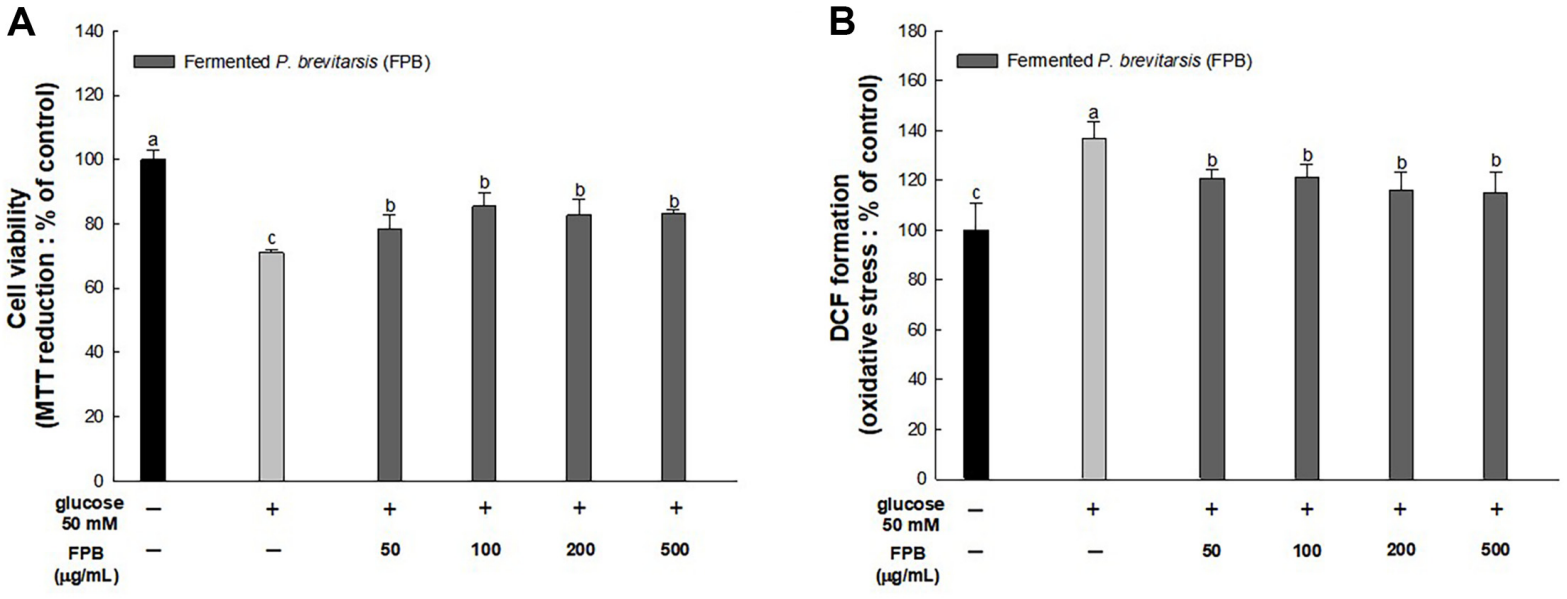
Effect of fermented *Protaetia brevitarsis* larvae (FPB) on high-fat diet (HFD)-induced diabetic mice. Fasting blood glucose (FBG) (**A**) oral glucose tolerance test (OGTT) (**B**) and area under the curve (AUC) in OGTT (**C**) Results are expressed as mean ± SD (*n* = 10). Data were statistically considered at *p* < 0.05, and different lowercase letters with superscripts represent statistical differences.

As shown in Fig. 3A, before FPB treatment, the FBG level showed an increase in the HFD group compared to the control group. Also, there was no significant difference between the FPB groups and the HFD group. However, significant differences were showed between the HFD group and the FPB from 2 weeks after FPB treatment. After 4 weeks of FPB treatment, FBG level was increased in the HFD group compared to the control group. However, the FPB groups was decreased compared to the HFD group.

As shown in Fig. 3B, OGTT was performed 0, 15, 30, 60, 90, and 120 min after glucose administration on the last day of the experiment (at 17^th^ week). In the control group, blood glucose levels increased at 15 min of glucose intake, but the levels gradually decreased thereafter. On the other hand, the HFD group was increased the blood glucose level until 30 min. However, the FPB groups decreased blood glucose level compared to the HFD group at 30 min after glucose intake.

As shown in Fig. 3C, the above experimental results were expressed as the AUC of OGTT was increased in the HFD group compared to the control group. However, the AUC of the FPB groups was decreased compared to the HFD group.

### Effect of FPB on Serum Biochemical Parameters

As shown in Table 1, the levels of serum biomarkers related to hepatic and renal toxicity are presented. HFD treatment (132.20, 202.40, 89.40, and 125.32 mg/dL) increased in the levels of TG, TCHO, HDLC, and LDLC compared to the control group. However, FPB consumption decreased the levels of TG, TCHO, HDLC, and LDLC compared with the HFD group.

HTR (%) (percentage of HDLC to TCHO) was decreased in the HFD group compared to the control group. However, the FPB groups increased the HTR compared to the HFD group.

As shown in Table 2, the levels of serum biomarkers related to hepatic and renal toxicity are presented. HFD treatment increased in the levels of GOT, GTP, LDH, TBIL, CRE, and BUN compared to the control group. However, FPB consumption decreased the levels of GOT, GTP, LDH, TBIL, CRE, and BUN compared with the HFD group.

### Effect of FPB on Changes in Weight of Tissues

As shown in Table 3, HFD treatment was induced increase of the weight of the liver, epididymal fat, perirenal fat, retroperitoneal fat, mesenteric fat, and total fat compared to the control group. However, FPB consumption was showed that the decrease of liver, epididymal fat, perirenal fat, retroperitoneal fat, mesenteric fat, and total fat compared to the HFD group.

### Effect of FPB on Antioxidant Parameters

**Fig. 4:**
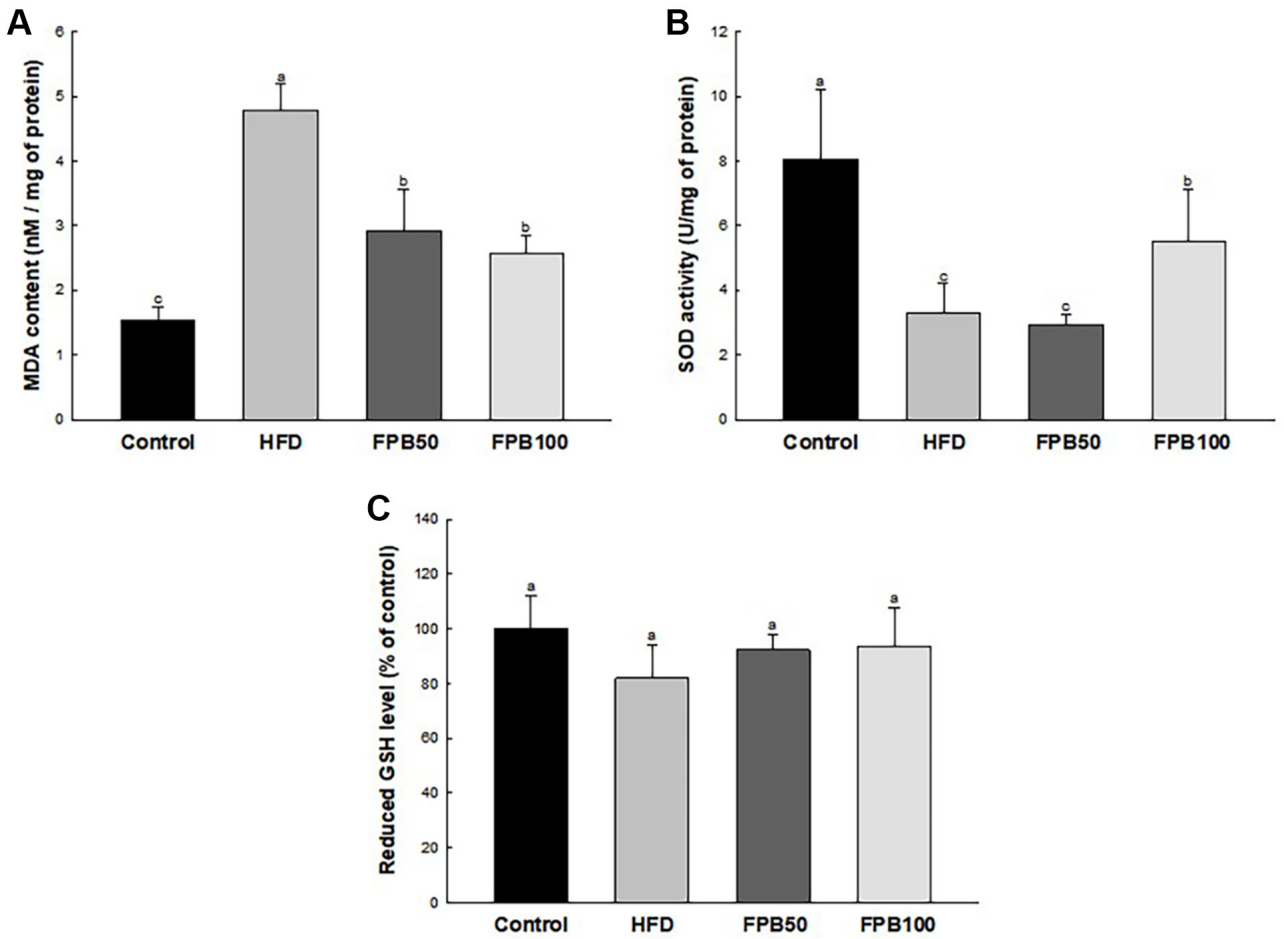
Effect of fermented *Protaetia brevitarsis* larvae (FPB) on antioxidant system in high-fat diet (HFD)-induced mice. Malondialdehyde (MDA) content (**A**) reduced glutathione (GSH) level (**B**) and superoxide dismutase (SOD) activity (**C**) in liver tissues. Results are expressed as mean ± SD (*n* = 7). Data were statistically considered at *p* < 0.05, and different lowercase letters represent statistical differences.

As shown in Fig. 4A, the MDA content in the liver tissues was increased in the HFD group compared to the control group. However, the MDA content of the FPB groups were reduced compared to the HFD group.

As shown in Fig. 4B, the SOD activity in the liver tissues was decreased in the HFD group compared to the control group. However, the SOD activity of the FPB 100 group were increased compared to the HFD group. There was no significant difference between the FPB 50 and the HFD groups.

As shown in Fig. 4C, the reduced GSH level in the liver tissues was decreased in the HFD group compared to the control group, but FPB groups were increased compared with the HFD group. However, there was no statistically significant difference between all groups.

### Effect of FPB on Mitochondrial Function

**Fig. 5:**
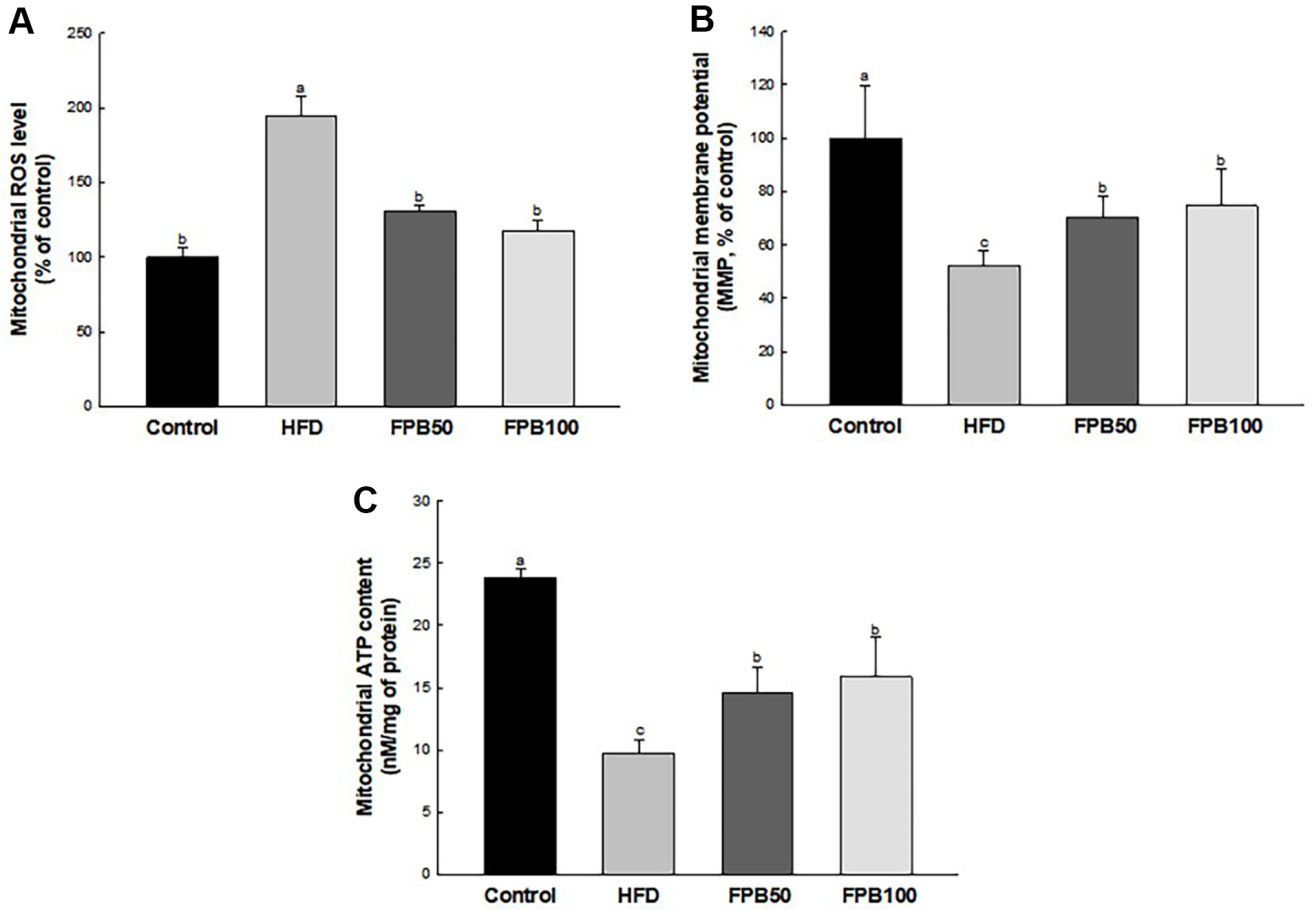
Effect of fermented *Protaetia brevitarsis* larvae (FPB) on mitochondrial function in high-fat diet (HFD)-induced mice. Mitochondrial reactive oxygen species (ROS) level (**A**) mitochondrial membrane potential (MMP) (**B**) and mitochondrial ATP content (**C**) in liver tissues. Results are expressed as mean ± SD (*n* = 5). Data were statistically considered at *p* < 0.05, and different lowercase letters represent statistical differences.

As shown in Fig. 5A, ROS level in the liver tissues was increased in the HFD group compared to the control group. However, the ROS level of the FPB groups were reduced compared to the HFD group.

As shown in Fig. 5B, MMP level in the liver tissues was reduced in the HFD group compared to the control group. However, the MMP of the FPB groups were increased compared to the HFD group.

As shown in Fig. 5C, ATP content in the liver tissues was decreased in the HFD group compared to the control group. However, the ATP level of the FPB groups were increased compared to the HFD group.

### Effect of FPB on Lipid Metabolism Pathway

**Fig. 6:**
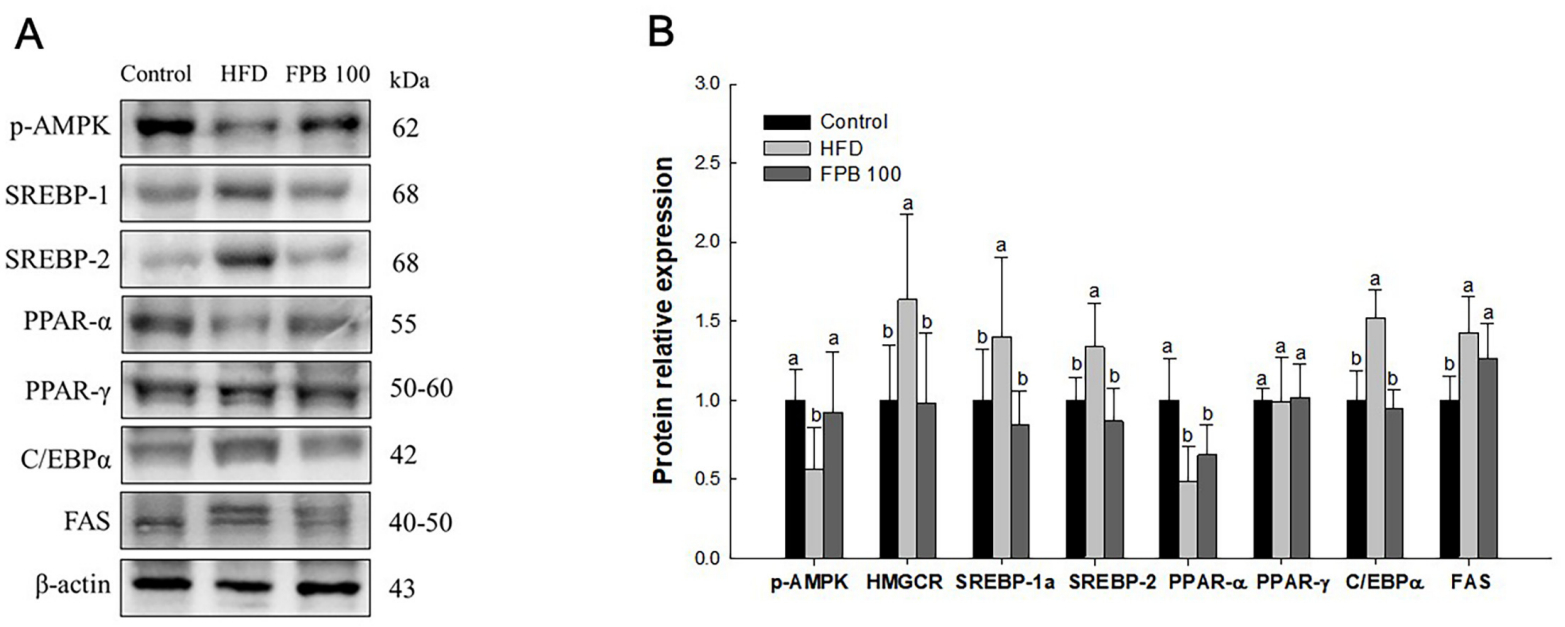
Effect of fermented *Protaetia brevitarsis* larvae (FPB) on lipid metabolism pathway in liver tissue of high-fat diet (HFD)-induced mice. Western blot band images (**A**). Relative expression level (**B**) of p-AMPK, HMGCR, SREBP-1a, SREBP-2, PPAR-α, PPAR-γ, C/EBPα, and FAS on the corresponding quantitation to β-actin. Results are expressed as mean ± SD (*n* = 3). Data were statistically considered at *p* < 0.05, and different lowercase letters represent statistical differences.

As shown in Fig. 6, the effect of FPB on the lipid metabolism was confirmed through western blot assay. The expression levels of p-AMPK and peroxisome proliferator-activated receptor (PPAR)-α in the liver tissues were decreased in the HFD group compared to the control group. However, the expression levels of p-AMPK and PPAR-α in the FPB 100 group were increased compared to the HFD group. However, there was no significant different between the HFD group and the FPB100 group in PPAR-α. The expression levels of HMGCR, sterol regulatory element-binding protein (SREBP)-1a, SREBP-2, CCAAT/enhancer binding protein (C/EBP) α, and fatty acid synthase (FAS) were increased in the HFD group compared to the control group. However, the expressions of HMGCR, SREBP-1a, SREBP-2, and C/EBPα, and FAS in the FPB 100 group were decreased compared to the HFD group. However, there was no significant different between the HFD group and the FPB100 group in FAS. Moreover, there was no significant difference in the expression level of PPAR-γ in all groups.

### Effect of FPB on Inflammatory Pathway

**Fig. 7:**
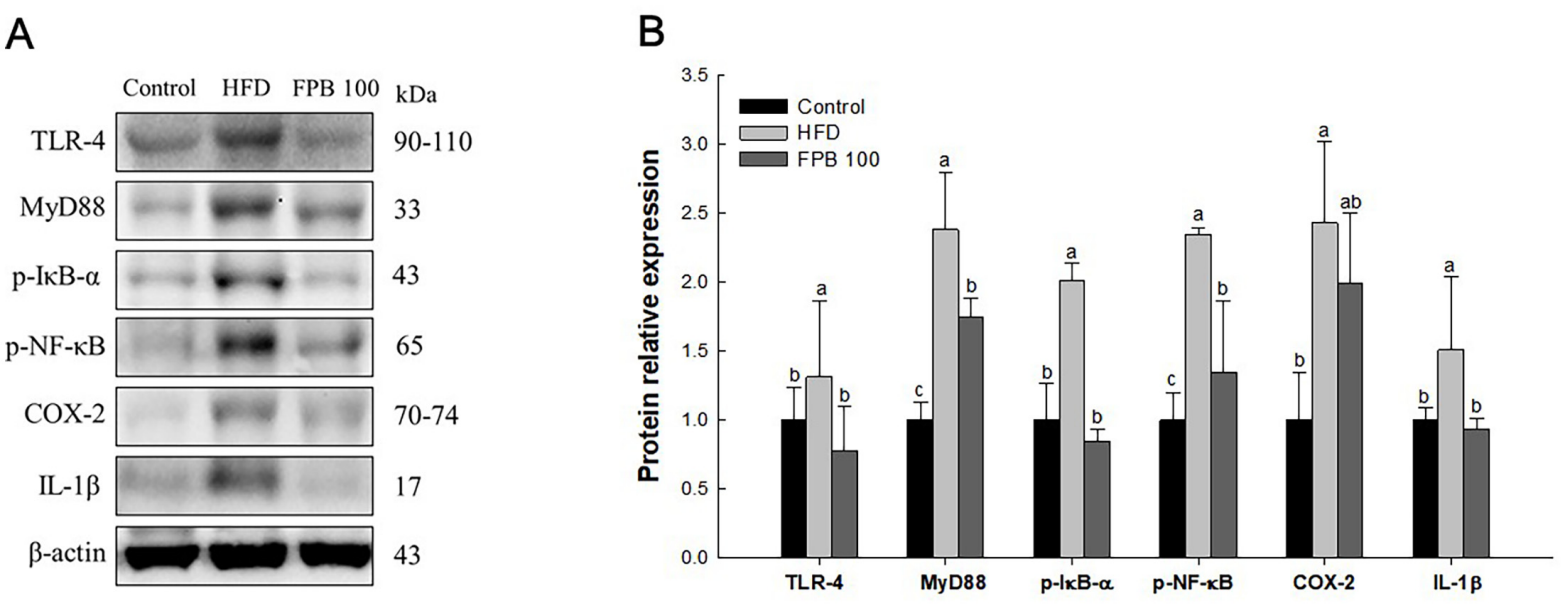
Effect of fermented *Protaetia brevitarsis* larvae (FPB) on inflammatory pathway in liver tissue of highfat diet (HFD)-induced mice. Western blot band images (**A**). Relative expression levels (**B**) of TLR-4, MyD88, p-IκB-α, p- NF-κB, COX-2, and IL-1β on the corresponding quantitation to β-actin. Results are expressed as mean ± SD (*n* = 3). Data were statistically considered at *p* < 0.05, and different lowercase letters represent statistical differences.

As shown in Fig. 7, the effect of FPB on the inflammatory pathway was confirmed through western blot assay. The expression levels of TLR-4, myeloid differentiation primary response 88 (MyD88), p-NF-κB inhibitor (p-IκB)-α, p-NF-κB, COX-2, and IL-1β were increased in the HFD group compared to the control group. However, the expression levels of TLR-4, MyD88, p-IκB-α, p-NF-κB, COX-2, and IL-1β in the FPB 100 group were reduced compared to the HFD group.

### Effect of FPB on Apoptosis Pathway

**Fig. 8:**
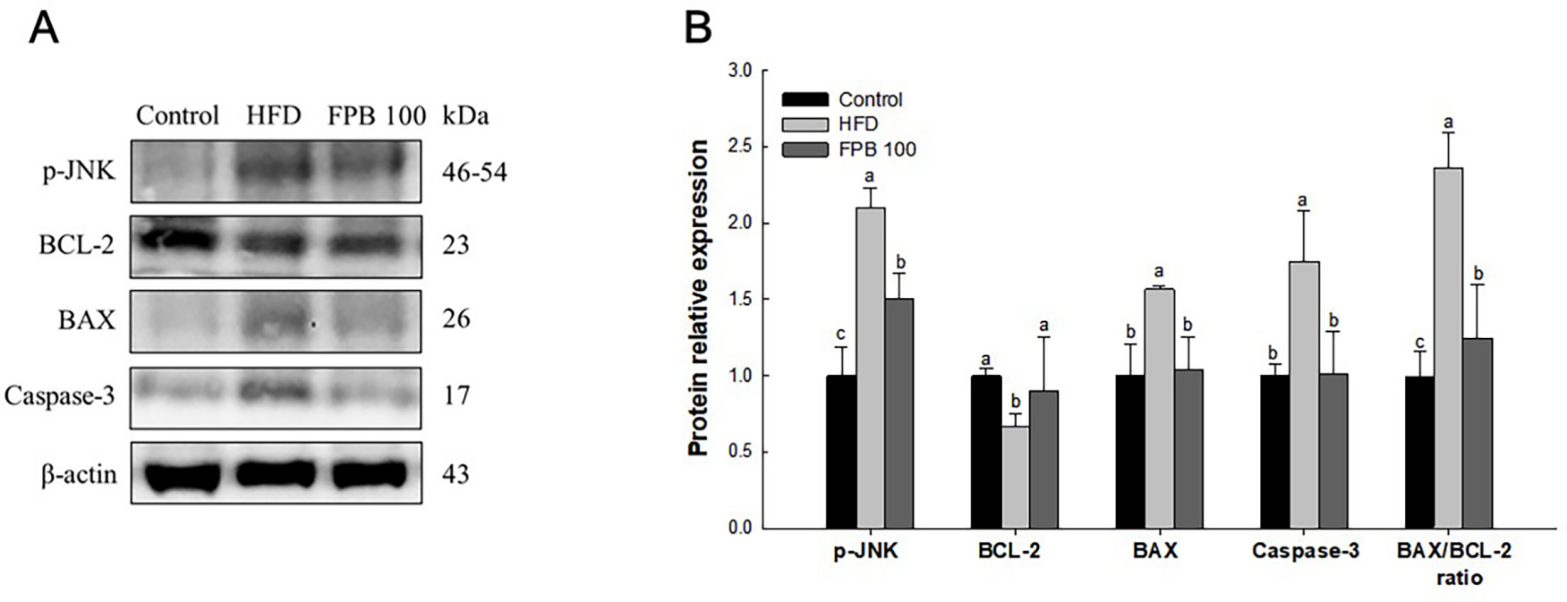
Effect of fermented *Protaetia brevitarsis* larvae (FPB) on apoptosis pathway in liver tissue of high-fat diet (HFD)-induced mice. Western blot band images (**A**). Relative expression levels (**B**) of p-JNK, BCL-2, BAX, caspase-3, and BAX/BCL-2 ratio on the corresponding quantitation to β-actin. Results are expressed as mean ± SD (*n* = 3). Data were statistically considered at *p* < 0.05, and different lowercase letters represent statistical differences.

As shown in Fig. 8, the effect of FPB on the apoptosis pathway was confirmed through western blot assay. The expression level of B-cell lymphoma (BCL)-2 in the HFD group was decreased compared to the control group. However, the expression level of BCL-2 was increased in the FPB 100 group compared to the HFD group. In contrast, the expression levels of p-c-Jun N-terminal kinase (p-JNK), BCL-2-associated X protein (BAX), caspase-3, and BAX/BCL-2 ratio were increased in the HFD group compared to the control group. However, the expression level of p-JNK, BAX, caspase-3, and BAX/BCL-2 ratio were decreased in the FPB 100 group compared to the HFD group.

## Discussion

Chronic intake of an HFD can lead to development of NAFLD, including hepatic steatosis, NASH, and cirrhosis [3]. Hyperglycemia and dyslipidemia induced by an HFD can cause metabolic disorders due not only to oxidative stress but also accumulation of lipid, mitochondrial dysfunction, and inflammation in the liver [24]. Therefore, early control of liver function before development of hepatic steatosis and NASH can be important in preventing the progression of NAFLD. Currently, studies have been conducted on the anti-obesity and anti-diabetic activities of *P. brevitarsis* larvae, but research on the mechanism of improving NAFLD is lacking. Consequently, this study evaluated the hepaprotective effect of FPB on HFD-induced liver injury mice.

T2DM and NAFLD are mutual risk factors, and the prevalence of NAFLD was found to be about two times higher in people with diabetes than in the general population [2]. Chronic hyperglycemia induced by HFD intakes can cause IR by destroying the metabolic function of pancreatic β cells [25]. Indeed, abnormal glucose tolerance is a predictor of steatohepatitis and fibrosis in NAFLD patients [26]. The characteristics of dyslipidemia in diabetic patients are summarized as an increase in TG and LDLC concentrations and a decrease in HDL cholesterol [27]. In the case of ordinary people, lipoprotein lipase (LPL) is not only activated by insulin but also inhibits hormone-sensitive lipase (HSL) activity, thereby preventing neutral fat from escaping from fat cells. In contrast, in T2DM patients with IR, HSL activity is increased, so TG cannot be stored in adipocytes and fatty acid accumulates in the blood in the form of very low-density lipoprotein [27]. Increased TG in the blood is converted by the cholesteryl ester transfer protein enzyme, which exchanges cholesterol and TG in HDL [28]. As a result, the content of HDLC decreases in the blood. In short, HFD can lead to T2DM with impaired glucose tolerance (IGT) and hyperlipidemia, which are major factors that increase the risk of developing NAFLD. Therefore, we conducted an indirect assessment of the anti-diabetic effect of FPB using OGTT and serum biochemical analysis. As a result, intakes of FPB reduced FBG and improved blood glucose spikes (Fig. 3) in this study. Moreover, FPB improved dyslipidemia by regulating serum lipid markers such as TG, TCHO, and LDLC (Table 1). In particular, HFD significantly reduced the concentration of HDLC in the serum, and FPB restored it. Similar to this, in a previous study, consumption of *P. brevitarsis* decreased plasma glucose levels and im-proved glucose tolerance in diabetic mice [28]. Moreover, it has been reported that *P. brevitarsis* reduced TG, TCHO, and LDLC level in serum of HFD and ethanol-induced obese mice [19, 29]. Although research on the anti-diabetic activity of edible insects is still lacking, various studies have been demonstrated the improvement effect of *P. brevitarsis* in HFD-induced obesity animal models [12, 29, 31]. Obesity and diabetes are closely related, and fat accumulation causes diabetes by interfering with the action of insulin [3, 4]. Therefore, the improved fasting glucose and glucose tolerance effects of FPB shown in this study are expected to contribute to the improvement of NAFLD.

Diabetes can affect various tissues, and oxidative stress has been identified as a major cause of its complications in liver disease [32]. Oxidative stress proceeds when the rate of oxidant production increases relative to the rate of oxidant scavenging. Due to diabetes, failure to store glucose in fat and muscle tissues causes hyperglycemia [27]. Excessive free glucose activates metabolism such as polyol, PKC, advanced glycation end products, and hexosamine pathways, which decreases the NADPH/NADP^+^ ratio and increases ROS production [32]. In normal cellular activity, O_2_^·-^ is decomposed into H_2_O_2_ by SOD, and H_2_O_2_ is further broken down into H_2_O and O_2_ by catalase. However, in the hyperglycemic state induced by chronic HFD intake, an oxidative imbalance occurs due to the excessive generation of ROS, surpassing the capacity of the antioxidant system [33]. This impairs the normal activity of the antioxidant system, reducing its ability to neutralize reactive oxygen species and exacerbating the accumulation of oxidative stress. Indeed, a decrease in the activity of the antioxidant defense system was confirmed in liver tissue damaged by hyperglycemia in previous studies [32-34]. Similarly, this study showed that HFD intake decreased SOD levels increased and MDA content, but FPB improved these antioxidant biomarkers (Fig. 4). In a previous study, *P. brevitarsis* larvae protected liver function by increasing SOD activity and reduced GSH content in ethanol-induced mice [19]. In addition, *P. brevitarsis* larvae suppressed the expression of oxidative enzymes such as iNOS and COX-2 in HFD-induced colitis mice [31]. Besides this, various *in vitro* and *in vivo* studies have reported the antioxidant activity of *P. brevitarsis* larvae [11-13]. Likewise, FPB showed the ABTS and DPPH radical scavenging activities and FRAP in this study (Fig. S2), which can support our animal studies. Therefore, it is presumed that the antioxidant activity of FPB shown in this study can improve liver injury caused by T2DM.

Mitochondrial dysfunction is defined as reduced numbers of mitochondria and mitochondrial biogenesis, decreased MMP and ATP, and accumulation of ROS [35]. Hyperglycemic conditions can produce excessive ROS and change the morphology of mitochondria [36]. Moreover, increased oxidative glucose metabolism may itself increase the production of ROS within mitochondria. A previous study has reported that HFD reduces ATP production by decreasing the activity of respiratory chain enzymes such as complexes I, II, IV, and V, indicating liver mitochondrial dysfunction [38]. Damaged mitochondria decrease MMP and activate the apoptotic pathway. Similarly, this study showed that MMP and ATP were decreased and mitochondrial ROS content was increased in HFD-induced NAFLD mice liver tissue. However, these results were effectively improved by FPB pretreatment (Fig. 5). According to Lee *et al*., *P. brevitarsis* larvae improved mitochondrial function through the protection of MMP balance in H_2_O_2_-induced C2C12 myoblast [39]. Furthermore, *P. brevitarsis* larvae recovered neurotoxicity by regulating MMP, ATP content, and ROS level in ethanol-induced mice [14]. In addition, *P. brevitarsis* larvae have been reported to contain various amino acids such as cysteine, methionine, and lysine, which are necessary for maintaining mitochondrial function [19]. These results confirmed the possibility that FPB can improve HFD-induced liver injury by protecting mitochondrial dysfunction from oxidative stress.

Hepatic steatosis is a representative event in NAFLD, and there are several mechanisms of HFD-induced fatty liver [40]. In normal metabolism, insulin secreted after a meal increases lipogenesis and reduces lipolysis and fatty acid oxidation processes in mitochondria. However, when mitochondria are damaged, β-oxidation does not occur properly, which inhibits fatty acid decomposition and leads to lipid accumulation in the liver [7]. On the other hand, AMPK is a protein kinase that is important in regulating intracellular lipid metabolism and maintaining energy balance [6]. Previous studies have reported that continuous HFD intake reduces AMPK activity [5, 41]. Inhibition of AMPK by HFD consumption increases the expression of HMGCR, which is involved in the early steps of cholesterol synthesis [6]. In conditions of IR, SREBP is activated and promotes the synthesis of cholesterol and fatty acids. There are three types of SREBPs (SREBP-1a, SREBP-1c, and SREBP-2) and two genes encode them and have different lipid metabolism functions [42]. They regulate fatty acid and cholesterol synthesis, increasing lipid profile [42]. Moreover, PPAR-γ and SREBPs stimulate the expression of FAS, leading to fatty acid synthesis [5]. In contrast, PPAR-α helps in lipolysis by regulating the expression of proteins involved in the transport of FFA and β-oxidation in liver and muscle tissues [43]. In summary, consumption of HFD causes fat storage in the liver by inhibiting the activity of AMPK, which regulates transcription factors involved in fatty acid and cholesterol biosynthesis. Therefore, activation of AMPK may be a potential target for preventing and treating NAFLD. In this study, HFD decreased expressions of activated AMPK and PPAR-α and increased expression levels of HMGCR, SREBPs, and FAS, but FPB improved this lipid metabolism disorder (Fig. 6). Moreover, FPB reduced weight of the liver and white adipose tissue (Table 3). *Tenebrio molitor* and *Allomyrina dichotoma* larvae prevented HFD-induced obesity by activating AMPK and inhibiting SREBP-1c, FAS, and acetyl-CoA carboxylase (ACC) [44, 45]. Furthermore, *P. brevitarsis* larvae have been reported to regulate lipogenesis-related factors such as adipocyte protein 2, FAS, and PPAR-γ [46]. Besides, *P. brevitarsis* larvae decreased weight of liver, kidney, lungs, spleen, and epididymal fat tissues in HFD-induced obesity mice [30]. Therefore, our results suggest that FPB can improve hepatic steatosis in HFD-induced NAFLD mice by increasing AMPK pathway.

NASH is a severe form of NAFLD, which is determined by various factors such as lipid deposition, inflammatory response, IR, and genetic factors [1]. It has been reported that hyperglycemia stimulates NADPH oxidase in the PKC pathway, thereby inducing TLR-2 and TLR-4 expression through PKC-α and PKC-δ, respectively [47]. Elevation of PKC-δ can induce MyD88-mediated activation of NF-κB by TLR-4 signaling [47]. After, IκB is phosphorylated and NF-κB translocate to the nucleus and becomes activated as phospho-NF-κB p-65 [14]. NF-κB is a major transcription factor in M1 macrophages and results in the expression of numerous inflammatory genes, including genes encoding TNF-α, IL-1β, IL-6, IL-12, and COX-2 [48]. A previous study reported that hepatic inflammation was induced by activating the NF-κB-inducing kinase/IκB kinase/NF-κB pathway in HFD fed-mice [49]. According to Jung *et al*., elevated fatty acids in plasma induce the activation of NF-κB through PKC and TLR-mediated pathways [50]. Taken together, hyperglycemia caused by HFD intake can activate PKC-mediated TLR/MyD88/NF-κB signaling to secrete pro-inflammatory cytokines. Therefore, downregulating NF-κB activation, which is involved in a wide range of cellular activities including regulation of inflammatory responses, immune regulation, and apoptosis, may prevent NASH development. In current study, the TLR-4/NF-κB pathway was activated by HFD, but FPB ameliorated this inflammatory response (Fig. 7). In a previous study, *P. brevitarsis* larvae ameliorated inflammation by inhibiting expressions of inducible iNOS, COX-2, IL-6, and p-NF-κB in lipopolysaccharide-stimulated RAW264.7 macrophages [13]. In addition, an immunomodulatory activity of *P. brevitarsis* larvae was identified by improving mitogen-activated protein kinase and NF-κB pathway [51]. According to Lee *et al*., *P. brevitarsis* larvae decreased IL-6 and TNF-α levels in serum of HFD-induced mice [46]. These results suggest that FPB can improve hepatitis by regulating TLR-4/MyD88/NF-κB signaling pathway caused by hyperglycemia in HFD-induced NAFLD mice.

Oxidative stress may contribute to the progression from simple steatosis to NASH as well as promote cytotoxicity. In diabetes, excessive production of ROS and NADH during glycolysis has been demonstrated to impair mitochondrial function, enhance cellular oxidative stress, and increase cell death [52]. Moreover, the underlying mechanism of NAFLD is a combination of excessive oxidative stress and abnormal inflammatory response, which damages hepatocytes by activating the transcription of pro-apoptotic genes [53]. In addition, HFD intake activates JNK in response to cell damage, stimulating apoptosis-related proteins [54]. The apoptotic process is regulated by the BCL-2 family proteins, a group of proteins that include both pro- and anti-apoptotic proteins [54]. When intracellular stress increases due to various causes, the pro-apoptotic protein BAX is activated and the permeability of the mitochondrial outer membrane increases [55]. In contrast, BCL-2, a representative anti-apoptotic protein, inhibits apoptosis by inhibiting the activity of BAX [7]. However, when the permeability of the mitochondrial membrane is increased by BAX activation, cytochrome c is released from the mitochondria into the cytosol [56]. The release of cytochrome c activates caspase-9, which stimulates caspase-3/7, leading to apoptosis [56]. Meanwhile, when hepatocytes are damaged, enzymes present in the liver can leak into the blood [57]. The initial and most important indicators for evaluating liver damage in T2DM are the levels of plasma alanine transaminase (ALT or GPT), aspartate transaminase (AST or GOT), γ-glutamyl transpeptidase (GGT), TBIL, and LDH [58]. According to a previous study, ALT and AST levels were increased in serum by 61.7% and 32.2% in HFD-induced NAFLD mice, and apoptosis was upregulated in liver tissue in both mice and human patients [60]. Consistent with these results, our results were showed that intake of HFD increased pro-apoptotic proteins, but FPB increased anti-apoptotic protein (Fig. 8). FPB also restored the HFD-induced increase in serum liver damage markers in this study (Table 2). In a previous study, *P. brevitarsis* larvae were improved neurotoxicity by regulating the protein kinase B/glycogen synthase kinase-3β pathway and BAX/BCL-2 ratio in ethanol-induced mice [14]. According to Lee *et al*., *P. brevitarsis* larvae inhibited H_2_O_2_-induced apoptosis in C2C12 cells as confirmed by flow cytometry [39]. Besides, it was reported that, protein hydrolysates derived from *P. brevitarsis* was reduced serum ALT, AST, alkaline phosphatase, bilirubin, CRE, and BUN levels in HFD-induced obesity mice [46]. Moreover, FPB showed a hepatoprotective effect on high glucose-induced cellular cytotoxicity (Fig. 1), which can support our animal study. Therefore, these results suggest that FPB can improve hepatoxicity by modulating the BCL-2 pathway caused by oxidative stress in HFD-induced NAFLD mice.

In conclusion, NAFLD is accompanied by simple hepatic steatosis and NASH and is closely related to obesity, T2DM, and dyslipidemia. Our results showed that HFD intake in-creased serum lipid levels, hepatic steatosis, NASH, and hepatic apoptosis. However, FPB revealed *in vitro* antioxidant activity and cytoprotective effects by increasing cell viability and reducing intracellular ROS in high glucose-induced HepG2 cells. *In vivo* results presented that FPB improved hyperglycemia in chronic HFD-induced IGT, as detected by FBG and OGTT tests. Moreover, FPB prevented hepatic oxidative stress by enhancing antioxidant parameters such as MDA content, reduced GSH level, and SOD activity. Besides, FPB enhanced hepatic mitochondrial function by regulating ROS level, MMP, and ATP content. These results suggest that FPB may have helped suppress lipotoxicity-mediated intrinsic apoptosis by regulating BCL-2 family proteins and caspase-3. In addition, FPB not only alleviated serum lipid levels and hepatotoxicity and nephrotoxicity biomarkers caused by hepatocyte damage but also reduced the weight of liver tissue and white adipose tissue. As a result, FPB ameliorated hepatic steatosis and hepatitis symptoms by activating AMPK/SREBPs pathway and inhibiting the TLR-4/NF-κB pathway. Although further studies on componential analysis are needed to better understand the functions of FPB, these findings demonstrated that FPB has the potential to be used as a preventive strategy or alternative medicine to ameliorate NAFLD.

## Supplemental Materials

Supplementary data for this paper are available on-line only at http://jmb.or.kr.



## Figures and Tables

**Table 1:** Effect of fermented *Protaetia brevitarsis* larvae (FPB) on triglyceride (TG), total cholesterol (TCHO), high density lipoprotein cholesterol (HDLC), low-density lipoprotein cholesterol (LDLC), and HDLC and TCHO ratio (HTR) in serum of high-fat diet (HFD)-induced mice.

	Control	HFD	FPB 50	FPB 100
TG (mg/dL)	112.20 ± 12.60^b^	132.2 ± 7.60^a^	102.8 ± 17.64^bc^	104.8 ± 12.5^c^
TCHO (mg/dL)	122.00 ± 13.47^d^	202.40 ± 9.63^a^	181.2 ± 20.92^b^	185.0 ± 23.90^b^
HDLC (mg/dL)	103.20 ± 3.96^d^	89.40 ± 7.96^c^	126.00 ± 11.85^b^	148.20 ± 15.32^a^
LDLC (mg/dL)	30.60 ± 2.43^d^	125.32 ± 12.43^a^	80.96 ± 18.31^b^	69.08 ± 6.39^c^
HTR (%)	85.41 ± 9.95^a^	44.23 ± 4.12^c^	70.65 ± 13.15^b^	80.86 ± 10.78^ab^

Results are expressed as mean ± SD (*n* = 7). Data were analyzed statistically at *p* < 0.05. Different lowercase superscript letters within rows indicate significant differences between groups.

**Table 2:** Effect of fermented Protaetia brevitarsis larvae (FPB) on glutamic oxaloacetic transaminase (GOT), glutamine pyruvic transaminase (GPT), lactate dehydrogenase (LDH), total bilirubin (TBIL) creatin (CRE), and blood urea nitrogen (BUN) in serum of high-fat diet (HFD)-induced mice.

	Control	HFD	FPB 50	FPB 100
GOT (U/L)	48.60 ± 1.67^d^	137.80 ± 24.57^a^	117.40 ± 17.76^b^	99.40 ± 20.65^c^
GPT (U/L)	29.80 ± 2.38^e^	260.60 ± 46.90^a^	153.40 ± 13.85^b^	102.60 ± 33.09^d^
LDH (U/L)	224.00 ± 38.06^e^	847.20 ± 118.06^a^	755.00 ± 128.23^b^	554.80 ± 69.15^d^
TBIL (mg/dL)	0.56 ± 0.11^b^	0.84 ± 0.21^a^	0.42 ± 0.04^c^	0.32 ± 0.04^d^
CRE (mg/dL)	0.15 ± 0.02^b^	0.21 ± 0.02^a^	0.12 ± 0.02^c^	0.10 ± 0.01^d^
BUN (mg/dL)	22.20 ±1.92^b^	26.08 ± 2.22^a^	17.30 ± 1.31^c^	17.26 ± 2.18^c^

Results are expressed as mean ± SD (*n* = 7). Data were analyzed statistically at *p* < 0.05. Different lowercase superscript letters within rows indicate significant differences between groups.

**Table 3:** Effect of fermented *Protaetia brevitarsis* larvae (FPB) on changes in tissues weight (unit: g) of highfat diet (HFD)-induced mice.

	Control	HFD	FPB 50	FPB 100
Liver	1.07 ± 0.05^d^	1.85 ± 0.25^a^	1.54 ± 0.10^b^	1.27 ± 0.20^c^
Epididymal fat	0.99 ± 0.11^d^	1.40 ± 0.26^a^	1.18 ± 0.04^bc^	1.12 ± 0.04^c^
Perirenal fat	0.06 ± 0.02^c^	0.37 ± 0.09^a^	0.27 ± 0.04^b^	0.25 ± 0.03^b^
Retroperitoneal fat	0.26 ± 0.02^c^	0.70 ± 0.11^a^	0.53 ± 0.11^b^	0.50 ± 0.08^b^
Mesenteric fat	0.33 ± 0.04^c^	0.94 ± 0.08^a^	0.73 ± 0.13^b^	0.72 ± 0.13^b^
Total fat	1.63 ± 0.49^c^	3.41 ± 0.41^a^	2.72 ± 0.39^b^	2.58 ± 0.33^b^

Results are expressed as mean ± SD (*n* = 7). Data were analyzed statistically at *p* < 0.05. Different lowercase superscript letters within rows indicate significant differences between groups.
