# Supplementary material for: Fermented Protaetia brevitarsis Larvae Alleviates High-Fat Diet-Induced Non-Alcoholic Fatty Liver Disease in C57BL/6 Mice via Regulation of Lipid Accumulation and Inflammation
Source: J Microbiol Biotechnol. 2025 Feb 10;35:e2409025. doi: 10.4014/jmb.2409.09025 (PMC11876019; doi:10.4014/jmb.2409.09025)
Supplement: Supplementary file 1 [file jmb-35-e2409025-supple.pdf]

J. Microbiol. Biotechnol. 2025. 35: e2409025
https://doi.org/10.4014/jmb.2409.09025

Supplementary Figures

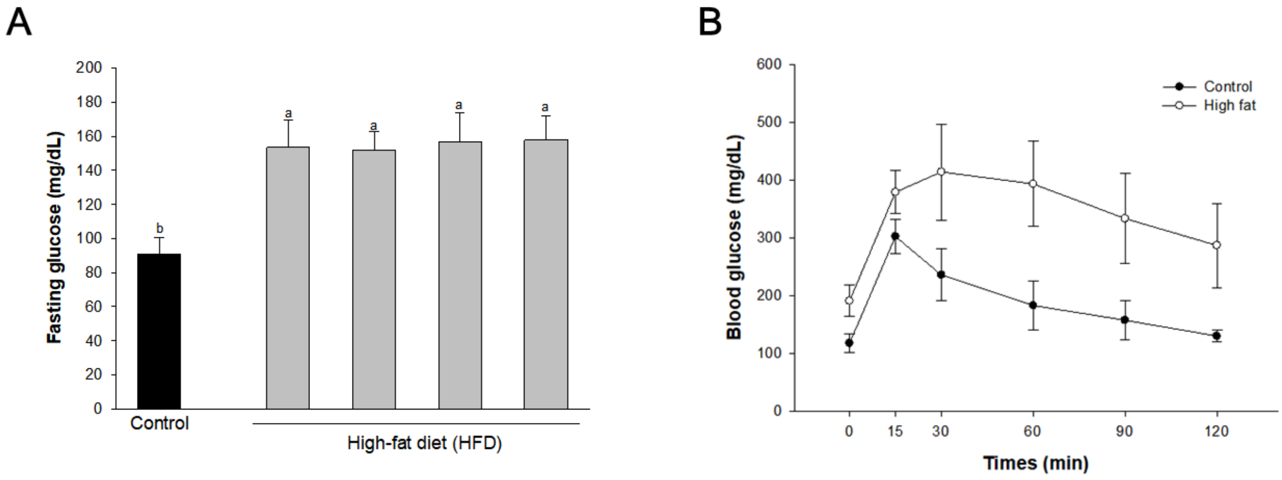

Fig. S1. Fasting blood glucose (FBG) (A) and oral glucose tolerance test (OGTT) of high-fat diet (HFD)-induced mice for 12 weeks. Results are expressed as mean ± SD (n = 10). Data were statistically considered at $p < 0.05$. Different lowercase letters with superscripts represents the statistical differences.

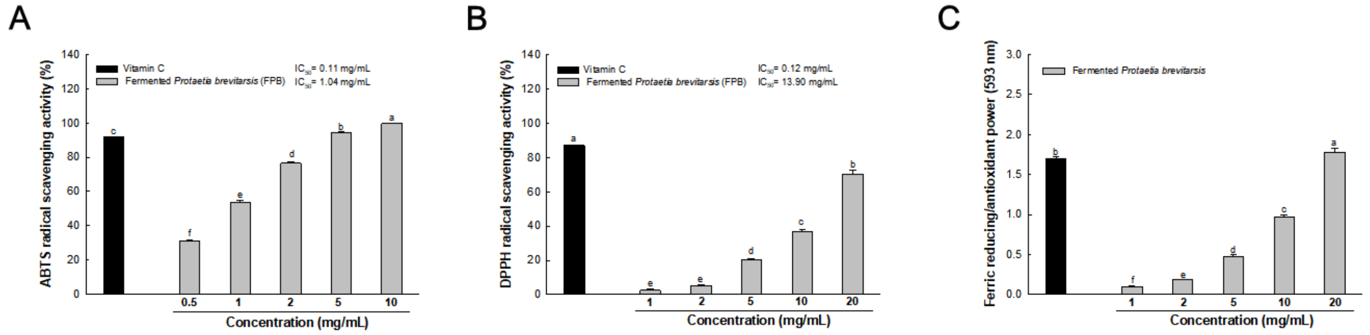

Fig. S2. ***In vitro*** **antioxidant effect of fermented *Protaetia brevitarsis* larvae (FPB) on ABTS radical scavenging activity** (**A**), DPPH radical scavenging activity (**B**), and FRAP (**C**). Results are expressed as mean ± SD (n = 3). Data were statistically considered at $p < 0.05$, and different lowercase letters with superscripts represent the statistical differences.
